# Role of β-Alanine Supplementation on Cognitive Function, Mood, and Physical Function in Older Adults; Double-Blind Randomized Controlled Study

**DOI:** 10.3390/nu15040923

**Published:** 2023-02-12

**Authors:** Ishay Ostfeld, Tavor Ben-Zeev, Amit Zamir, Chagai Levi, Yftach Gepner, Shmuel Springer, Jay R. Hoffman

**Affiliations:** 1 School of Health Science, Ariel University, Ariel 40700, Israel; 2 Department of Epidemiology and Preventive Medicine, School of Public Health, Sackler Faculty of Medicine, Sylvan Adams Sports Institute, Tel Aviv University, Tel Aviv 6997801, Israel

**Keywords:** supplementation, pattern recognition, nutrition, health, aging

## Abstract

This study investigated 10 weeks of β-alanine (BA) supplementation on changes in cognitive function, mood, and physical performance in 100 older adults (70.6 ± 8.7 y). Participants were randomized into a BA (2.4 g·d^−1^) or placebo (PL) group. Testing occurred prior to supplementation (PRE), at the midpoint (MID), and at week-10 (POST). Participants completed cognitive function assessments, including the Montreal cognitive assessment (MOCA) and the Stroop pattern recognition test, at each testing session. Behavioral questionnaires [i.e., the profile of mood states, geriatric depression scale (GDS), and geriatric anxiety scale (GAS)] and physical function assessments (grip strength and timed sit-to-stand) were also conducted. No difference between groups was noted in MoCA scores (*p* = 0.19). However, when examining participants whose MOCA scores at PRE were at or below normal (i.e., ≤26), participants in BA experienced significant improvements in MOCA scores at MID (13.6%, *p* = 0.009) and POST (11.8%, *p* = 0.016), compared to PL. No differences were noted in mood scores, GAS, or any of the physical performance measures. A significant decrease was observed in the GDS for participants consuming BA but not in PL. Results suggested that BA supplementation can improve cognitive function in older adults whose cognitive function at baseline was at or below normal and possibly reduce depression scores.

## 1. Introduction

The benefits of β-alanine supplementation have been well-established for the past 15+ years [1,2]. Supplementation with β-alanine increases muscle carnosine concentrations, which enhances the muscle’s ability to buffer increases in hydrogen ion formation during high-intensity exercise [3]. Early studies focusing on high-intensity exercise were able to demonstrate consistent efficacy of β-alanine ingestion and improvements in exercise performance. By increasing muscle buffering capacity, anaerobic athletes were able to delay fatigue and maintain high-intensity exercise for a prolonged period of time [4,5,6]. As research accumulated on the use of β-alanine as an ergogenic aid, subsequent meta-analyses have indicated that β-alanine is efficacious at increasing exercise capacity [2]. In addition, understanding of the physiological role that elevations in carnosine have in tissue also resulted in the hypothesis that β-alanine supplementation may have a role as an antioxidant [7]. In studies examining the efficacy of β-alanine supplementation on soldiers in special operation units, the ability to improve physical function was observed, but the investigations also indicated that those soldiers that supplemented with β-alanine also experienced significant improvements in cognitive function during periods of high stress (e.g., performing mathematical equations on a firing line and being able to expeditiously handle a misfire and continue to shoot quickly and accurately) [8,9]. This suggested that β-alanine was potentially acting in tissue besides skeletal muscle.

Animal investigations subsequently demonstrated that β-alanine can cross the blood–brain barrier and increase carnosine content in all brain regions in both young and older rats [10,11]. In addition, elevations in brain carnosine were associated with maintaining brain-derived neurotrophic factor expression (BDNF) during both fear [11] and blast exposure [12] stressors. Animals provided with β-alanine experienced reduced brain inflammation, reduced anxiety, and maintained spatial memory compared to animals exposed to these stimuli but given a placebo. These studies suggested that β-alanine supplementation could impact neural tissue and potentially promote brain health. As humans age, increased oxidative stress and inflammation in the brain are found to be associated with cognitive decline and a greater risk for neuroinflammatory disease [13,14].

The benefits of β-alanine supplementation on functional performance in older adults have been reported in several investigations. These human studies have demonstrated that supplementing with β-alanine can delay fatigue, increase strength, and improve functional performance (e.g., sit to stand) [15,16]. Limited research has indicated that β-alanine supplementation in older adults is also associated with improved executive function [17]. Although increasing tissue carnosine levels have been reported to improve health in older adults [18,19,20], there is only limited scientific evidence supporting the efficacy of β-alanine as a therapeutic intervention to improve cognitive function and memory in this population. Considering the anti-inflammatory and antioxidant effects attributed to increased tissue carnosine levels, the purpose of this study was to examine the effect of β-alanine supplementation on cognitive function, mood, and physical function in sedentary older adults. The study’s hypothesis is that 10 weeks of β-alanine supplementation can improve cognitive function and improve executive function in older adults.

## 2. Materials and Methods

### 2.1. Participants

One hundred older (between the ages of 60–80) adult men (n = 29) and women (n = 71) volunteered to participate in this double-blind, randomized controlled study. Recruitment occurred via word-of-mouth discussions with various community center leaders allowing the investigative team to provide a talk to prospective participants. Once a participant expressed an interest in participating in the study, they were interviewed prior to study enrollment to ensure that they were not supplementing with β-alanine (BA) in the previous 6 months prior to the study period. In addition, it was also determined whether they had been previously diagnosed with dementia, neurocognitive function disease (i.e., Alzheimer’s or Parkinson’s disease), or any other medical condition that would preclude participation in the study. A participant who had any underlying neurological condition was not eligible to enroll in the study. All interviews were conducted by a licensed physician. The study protocol was approved by the Ethics Committee at Ariel University (AU-HEA-YO-20201117). Participants were not permitted to use any additional nutritional supplements for at least 6-weeks prior to the study. Screening for supplement use and medical history was accomplished during the interview with the study physician. This study was registered as a clinical trial with the Israel Ministry of Health (MOH_2021-09-30_010279).

### 2.2. Study Protocol

Testing took place at various community centers across the country. Participants met with the study team on three separate occasions. At the first visit (PRE), participants received an explanation about the study, provided their informed consent, and were randomized into either the BA (15 men and 35 women; 68.4 ± 6.1 y; 168.5 ± 10.4 cm and 77.9 ± 16.2 kg) or the placebo group (PL; 14 men and 36 women; 70.8 ± 9.3 y; 165.0 ± 9.1 cm and 71.4 ± 13.8 kg). Before taking their first supplement, participants completed several tests consisting of written questionnaires to assess cognitive function and mood and a computer-based exam to measure cognitive and executive function. In addition, a series of physical function tests were also performed. At the end of the testing session, participants were provided with a bottle containing the supplement or placebo. They were given instructions on how and when to take the supplement and that they should bring the bottle back to the community center at the next testing session. Testing was also conducted at weeks 5 (MID) and 10 (POST). At MID, participants provided their supplement bottles and received new bottles. To determine participant compliance, the number of capsules remaining in the bottle were counted and recorded. Participants completed the same testing protocol in the same order as completed during PRE. The same procedure was completed at POST, except that participants were not given a new bottle.

### 2.3. Supplement Protocol

Participants were instructed to consume two tablets (600 mg apiece) twice per day (total supplement or placebo consumed per day was 2.4 g) for the duration of the 10-week study. Participants were instructed to consume the supplement with their regular meals. The supplement and placebo were identical in appearance. The BA tablet was a sustained-release formulation, and the PL tablet consisted of the same ingredients but contained hydroxypropyl methylcellulose in place of the BA. The BA and PL tablets were provided by Natural Alternatives International (Carlsbad, CA, USA). Supplement compliance was tracked by counting the number of tablets remaining in the bottles at MID and POST testing. To remain in the final analysis, participant compliance was set at 80%.

### 2.4. Cognitive and Mood Measures

Cognitive function was evaluated with the Montreal Cognitive Assessment (MoCA) tool [21]. The MoCA is a simple, stand-alone cognitive screening that focuses on several cognitive domains, including executive function, memory, attention, language, abstraction, delayed recall, and orientation. It was developed as a screening instrument for the detection of mild cognitive impairment in older adults. Research has indicated that it has excellent test-retest reliability and positive and negative predictive values for mild cognitive impairment [22]. There are well over 1000 publications that have examined MoCA in older adults, with reliability and validity established in more than 40 languages, including Hebrew [23].

Participants also performed the Stroop test [24]. The Stroop test is a computerized test of attention and both cognitive and executive function. The Stroop test is divided into two stages: the learning stage and the test stage. During the test, four different words (red, blue, green, and yellow) appearing in four different colors were presented to the participants on a computer screen. Participants were asked to respond to the color of the word and not to its meaning. For example, the word red in blue color would be selected as blue. In the learning stage, 16 words were presented to the participant, but their scores were not recorded. In the test stage, 240 words were presented to the participant. The participant’s reaction time from appearance to decision was recorded. In addition, their success rate (e.g., how many correct answers/total questions) and speed-accuracy (e.g., reaction time/success rate) were determined. The validity and reliability of the computer-based Stroop test have been previously demonstrated in older adults [25].

The Profile of Mood States (POMS) was performed to assess the mood of the participants [26]. The POMS consists of 58 words or phrases in a Likert format questionnaire that provides measures of tension, depression, anger, vigor, fatigue, and confusion. A total mood score (TMS) was calculated by subtracting vigor from the sum of the five other negative measures. Participants were asked to respond to each word on how they felt for the past week. Measures of consistency ranging between 0.85 and 0.95 and test-retest reliability estimates ranging between 0.65 and 0.74 have been previously reported for the POMS instrument [26]. In addition, participants were also asked to complete the geriatric depression scale (GDS) and the geriatric anxiety scale (GAS). The GDS is a 15-question questionnaire in which participants were asked to respond by answering “Yes” or “No” to questions in reference to how they felt over the past week [27]. Of the 15 items, 10 indicated the presence of depression if answered positively, while the other five items indicated depression if answered negatively. Scores between 0–4 are considered normal; scores between 5–8 are considered to be representative of mild depression; scores between 9–11 are considered to be representative of moderate depression; and scores between 12–15 are considered to be indicative of severe depression. The GDS has been reported to have a 91% sensitivity when evaluated against diagnostic criteria [27]. The validity and reliability of this tool have been supported through both clinical practice and research [28]. The GAS is a 10-item self-report measure used to assess anxiety symptoms among older adults. Participants were asked to indicate how often they have experienced each symptom during the last week, answering on a 4-point Likert scale with verbal anchors ranging from “Not at all” to “All the time”. GAS items were derived from the broad range of anxiety disorder symptoms found in the Diagnostic and Statistical Manual of Mental Disorders [29]. The reliability and validity of the GAS for the quantitative assessment of anxiety symptoms in a diverse community and clinical samples of older adults have been previously established [30].

### 2.5. Performance Assessments

Participants were assessed on physical function measures of both the upper and lower body. A hand grip dynamometer test was used to assess upper body muscular strength, and a sit-to-stand test was used to measure lower body functionality [16]. During the hand grip assessment, participants stood with their feet shoulder-width apart, and the dynamometer (Jamar 5030 J1, Sammons Preston, Inc., Bolingbrook, IL, USA) held in their dominant hand. The dynamometer was adjusted so that the palm side of the grip was at the palm and lined up between the joint of the medial and distal phalanges. The dominant arm was placed close to the body (e.g., adduction) with the elbow bent at 90°. Participants were asked to squeeze the handle as hard as possible for 3–5 s. Three trials were performed, with 30 s of rest provided between trials. The maximum value attained was recorded. Test-retest reliability for the hand grip test was determined by using 20 participants in the placebo group measured 5 weeks apart. The ICC (3,1) was 0.956 (SEM = 2.8 kg).

During the sit-to-stand assessment, participants performed five consecutive full chair rises from a seated position in a standard armless chair. Participants were instructed to stand as fast as possible from the sitting position with their arms folded across their chest. A hand-held stopwatch was used to time test. The stopwatch began on the participant’s initial movement and stopped as the participant stood upright following the fifth repetition. The time to perform all five repetitions was recorded. In addition, each participant wore a belt around their waist, which was connected to a Tendo™ Power Output Unit (Tendo Sports Machines, Trencin, Slovak Republic). The Tendo™ unit consists of a transducer attached to the end of the belt, which measures linear displacement and time. Subsequently, the velocity of each movement was determined, and power was calculated. In addition to recording the highest power attained, the power output of all five repetitions was averaged (e.g., mean power), and the lowest power output attained was divided by the highest power output to provide a fatigue index. Test-retest reliability for the sit-to-stand test was determined by using 20 participants in the placebo group measured 5 weeks apart. The ICC (3,1) was 0.773 (SEM = 1.49 s).

### 2.6. Statistical Analysis

Prior to analysis, all data were assessed to ensure normal distribution, homogeneity of variance and sphericity. If sphericity was violated, a Greenhouse–Geisser correction was applied. Statistical evaluation of cognitive and performance changes was accomplished using repeated analysis of variance (ANOVA). In the event of a significant F ratio, least significant difference (LSD) post-hoc pairwise comparisons were used to examine the differences. The POMS, GDS and GAS were evaluated on an ordinal scale; thus, the Friedman non-parametric test was used to analyze group differences. All statistical analyses were analyzed using SPSS v27 software (SPSS Inc., Chicago, IL, USA), and an alpha level of *p* ≤ 0.05 was used to determine statistical significance. All data are reported as mean ± SD.

## 3. Results

**Figure 1:**
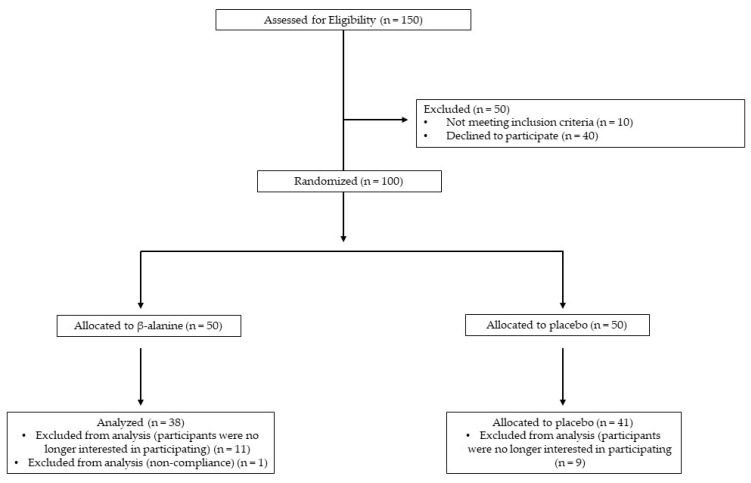
Study Flow Chart of Recruitment and Participant Analysis.

A total of 79 participants completed the study (BA = 38 and PL = 41). Of the participants who completed the study, the mean compliance was 92.0 ± 7.4%. No adverse events were reported, and of the 21 participants that did not complete the study, only one was dropped for non-compliance; the remaining dropouts decided not to continue their participation voluntarily. Figure 1 provides a study flow chart of the recruitment and final participant analysis.

### 3.1. Results of the MoCA Assessment

**Figure 2:**
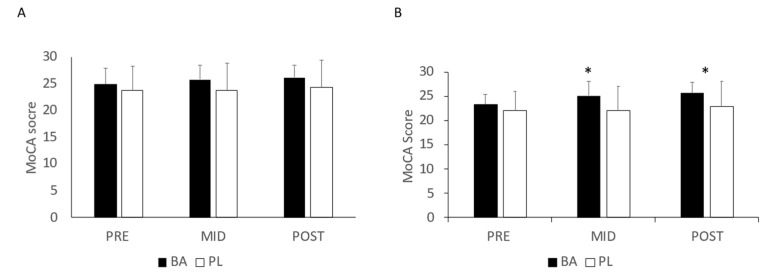
MoCA scores. (**A**) all participants; (**B**) Only participants with a MoCA score ≤ 26; * = Significant difference between groups. BA = β-alanine; PL = Placebo.All results are presented as mean ± SD.

The results of the MoCA assessment can be seen in Figure 2A,B. When comparing all participants in BA and PL that completed the study, a significant improvement in MoCA scores was noted with both groups combined (F = 5.4, *p* = 0.005). These improvements were noted from PRE to POST. However, no significant interaction between the groups was observed (F = 1.7, *p* = 0.190) (Figure 1A). When MoCA scores were examined in participants with borderline or below normal MoCA scores (i.e., ≤ 26), a significant interaction was found (F= 3.37. *p* = 0.042) (Figure 1B). Post-hoc analysis revealed that MoCA scores were significantly greater for BA than for PL at both MID (*p* = 0.009) and POST (*p* = 0.016).

### 3.2. Results of the Stroop Test

**Figure 3:**
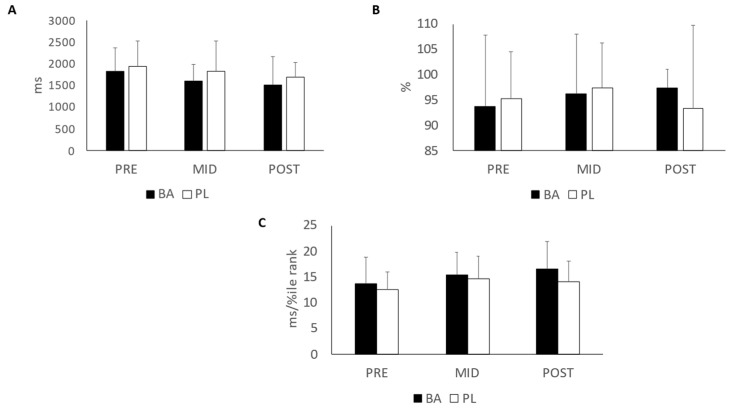
Stroop Test. (**A**) Reaction time; (**B**) Success rate; (**C**) Speed-Accuracy. BA = β-alanine; PL = Placebo. All results are presented as mean ± SD.

The results of the Stroop test can be seen in Figure 3A–C. Comparisons between BA and PL for all participants revealed a significant difference in time for all groups combined (F = 8.83, *p* < 0.001). Significant improvements in reaction time were noted from PRE to MID (*p* = 0.027) and from PRE to POST (*p* < 0.001). However, no significant interactions between BA and PL were noted in reaction time (F = 0.343, *p* = 0.711). No significant change from PRE was noted in success rate for both groups combined (F = 1.33, *p* = 0.269), but a trend was noted in the interaction between the groups (F = 2.525, *p* = 0.091). The results of the combination of reaction time and success rate, reported as speed-accuracy, revealed a significant improvement with both groups combined (F = 15.1, *p* < 0.001) but no significant interaction between the groups (F = 1.49, *p* = 0.23). The improvement in speed-accuracy was noted between PRE and MID and between PRE and POST (*p*’s < 0.001). When the raw score achieved in the speed-accuracy calculation was converted to a T score, participants that were below the population mean (BA = 16 and PL = 17) in this study (T < 50) were analyzed separately. This additional analysis revealed no between group interactions (*p*’s > 0.05) in reaction time, success rate or speed-accuracy.

### 3.3. Results of the Behavioral Measures

**Figure 4:**
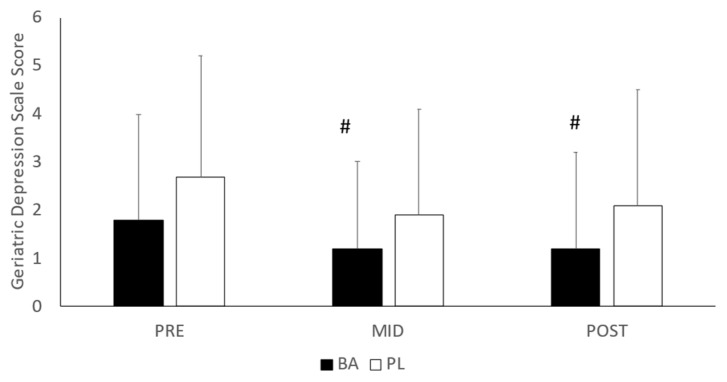
Geriatric Depression Scale. BA = β-alanine; PL = Placebo; # = Significantly different from PRE. All results are presented as mean ± SD.

**Figure 5:**
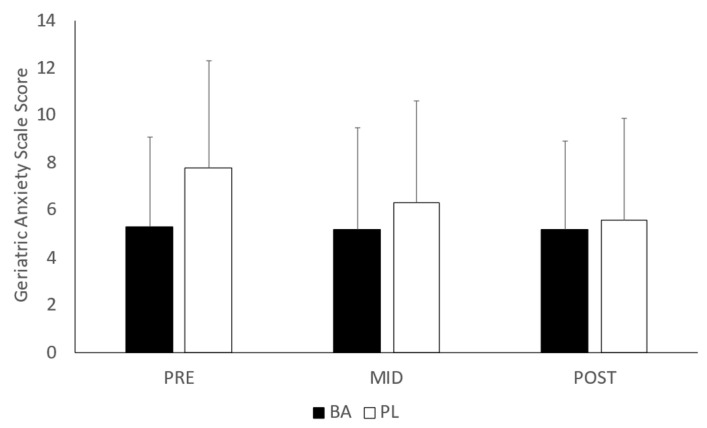
Geriatric Anxiety Scale. BA = β-alanine; PL = Placebo; All results are presented as mean ± SD.

The results of the POMS assessments are depicted in Table 1. No significant interactions were noted between the groups in tension (*p* = 0.571), depression (*p* = 0.559), anger/hostility (*p* = 0.103), vigor (*p* = 0.101), fatigue (*p* = 0.164) or confusion (*p* = 0.131). In addition, no differences between BA and PL were noted in TMS (*p* = 0.131). Examination of the GDS and GAS (see Figure 4 and Figure 5, respectively) revealed a significant group difference in GDS (*p* =0.037) but only a trend toward a difference in GAS (*p* = 0.096). Post-hoc analysis for GDS indicated that the mean rank for depression scores was significantly lower at MID (*p* = 0.012) and POST (*p* = 0.002) compared to PRE in the BA group. No significant differences from PRE were noted in PL.

### 3.4. Results of the Physical Performance Assessments

The results of the performance assessments can be seen in Table 2. No significant changes in body mass (F = 0.329, *p* = 0.721) were observed in either group. Analysis of changes in hand grip strength revealed a trend towards an improvement with both groups combined (F = 2.933, *p* = 0.66) but no significant interaction (F = 0.258, *p* = 0.773). Examination of performance in the sit-to-stand test revealed a significant main effect for time (F = 22.1, *p* < 0.001) with both groups combined but no significant interaction (F = 0.320, *p* = 0.727). Improvements were observed from PRE to MID (*p* < 0.001) and from MID to POST (*p* = 0.005). Although no significant main effect was noted for peak power during the sit-to-stand assessment (F = 1.103, *p* = 0.335), a trend for an interaction was observed (F = 2.554, *p* = 0.093. No main effects for time nor interactions were observed in mean power output (F = 1.46, *p* = 0.235 and F = 1.312, *p* = 0.272, respectively) or fatigue rate (F = 0.326, *p* = 0.723 and F = 1.081, *p* = 0.342, respectively) during the five sit-to-stand repetitions.

## 4. Discussion

The results of this study indicated that BA supplementation in older adults, whose cognitive function was at or below normal according to the MoCA normative results, was able to provide a significant benefit following 5 and 10 weeks of supplementation. In addition, a decrease in depression was also observed. No changes in mood, anxiety or physical performance assessments were noted.

Only one previous study has examined the effects of BA on cognitive function in older adults. Furst and colleagues [17] showed that performance on the Stroop test, a measure of executive function, was maintained following endurance exercise in middle-aged men and women (60.5 ± 8.6 y) following 28 days of BA supplementation (2.4 g·d^−1^). Solis et al. [31], who examined the effect of 28 days of BA supplementation (6.4 g·d^−1^) in trained men and women cyclists in their 4th decade of life (37.8 ± 8 y), found no change in the Stroop test following a 20 km time trial. To the best of our knowledge, the present study is the first to demonstrate that BA supplementation improves cognitive function in older adults, whose baseline cognitive function was borderline normal to below normal. Furthermore, in contrast to the study by Furst et al. [17], these changes were observed in participants who did not respond to any stressful event. The contrasting results of both the present study and the Furst et al. study [17] compared to Solis et al. [31] may be related in part to the age of the participants. Both studies showing cognitive benefits to BA supplementation examined middle-aged to older adults. A decline in cognitive function appears to become more prevalent as individuals age [14]. Although the mechanism associated with cognitive decline may be broad, a number of studies have suggested that elevations in neuroinflammation and oxidative stress are thought to contribute to this decline [13,14]. Considering that there is no evidence indicating that BA supplementation is ergogenic for cognitive improvements, the potential benefits observed in older adults with borderline normal or below cognitive function is the result of potential anti-inflammatory or antioxidant action resulting from carnosine elevation in brain tissue.

To date, there is limited evidence that BA supplementation can increase carnosine content in the brain of humans. Two separate studies examining the effect of BA supplementation and changes in carnosine content in the brain using magnetic resonance spectroscopy were unable to see any changes following 4 weeks of supplementation using 6.0 and 6.4 g·d^−1^ [9,31]. However, this may be a function of technological limitations, as several animal studies have demonstrated that BA supplementation can increase carnosine content in the brain tissue of both older and younger animals [10,11]. Studies reporting increases in brain carnosine content were associated with reductions in brain inflammation and increases in brain neurotrophin expression during exposure to various stressors [11,12]. However, in a study of older animals that were supplemented with BA, an increase in brain carnosine content was observed [10], but no attenuation of age-related inflammation was noted, and no change in learning was observed [32]. It was suggested that BA might be more effective in increasing resiliency but not effective in reversing age-related inflammation. The results of the present study provide evidence that BA can improve cognitive function in older adults whose cognitive abilities are borderline or below normal. However, the mechanism remains speculative.

The results of the Stroop test indicated a trend toward improvement with BA supplementation. In contrast to Furst et al. [17], we did not examine changes following an exercise stress; thus, it is difficult to compare the results of the two investigations. This trend was noted in all participants who consumed BA. A closer examination of performance on the Stroop test (i.e., examination of participants that scored below a T score of 50) was unable to shed any additional benefit in improvements of executive function. The differences in benefits observed between the MoCA and Stroop test may also be related to differences in the brain location responsible for their performance. The hippocampus is thought to be responsible for memory [33], whereas executive function is primarily controlled by the frontal cortex [34]. Although the MoCA includes assessments of executive function, the focus of the Stroop test is primarily on executive function, which may have contributed to these differences. It also should be acknowledged that normal Stroop performance is considered to be a T score above 40 [35]. We used 50 as a level of differentiation due to the small number of participants with a T score of 40 or below (n = 13). Further research is needed to provide additional insight into how BA intervention may affect these two measures of cognitive assessment differently.

Previous studies in animal models indicated that BA supplementation was able to reduce anxiety levels when animals were exposed to various stressors [11,12]. These changes were thought to be associated with the maintenance of brain-derived neurotrophic factors as a result of exposure to stress. In human studies, Hoffman and colleagues [36] suggested that BA supplementation was able to enhance focus in college football players during their condition programs. Although no changes in any of the mood states evaluated with the POMS or in the TMS were observed, a significant decrease was noted in the GDS in participants consuming BA but not in the PL group. No changes were seen in either group for GAS. These results were similar to Varanoske et al. [37], who reported significant decreases in depression in individuals supplementing with BA prior to simulated military operation stress. Improvements in mood (e.g., depression and anxiety) resulting from BA supplementation have been suggested to be related to changes in BDNF expression in the hippocampus [10,11,38]. Varanoske et al. [37] did not detect any changes in circulating BDNF concentrations in their study and indicated that blood concentrations do not reflect changes in BDNF expression in the brain.

In contrast to previous studies examining the use of BA by older adults [15,16], no significant changes were observed in any of the physical function measures. This was likely related to the lack of a training program in this study compared to the other investigations that demonstrated a positive effect of BA supplementation in conjunction with a physical training program.

There were several limitations to this study. Most studies examining the benefits of BA supplementation have examined the combined effects of BA supplementation with exercise. However, considering that most older adults that supplement do so for reasons related to health and wellness and not to enhance exercise performance [39], the format used in the present study appears to be relevant. Regardless, there was a 21% dropout or non-compliance among the participants. Considering that the prevalence of supplement use among Israel’s older adult community appears to be lower than that in North America or elsewhere [39,40], it is possible that the lack of a “culture” of supplement use may have limited sustained participation in the study in this particular population.

In conclusion, the results of this study indicated that BA supplementation enhanced cognitive performance in older adults whose baseline cognitive levels were borderline to below normal. In addition, taking BA supplementation was able to reduce symptoms of depression.

## Figures and Tables

**Table 1:** Profile of Mood States.

Group	Time	Tension	Depression	Anger/Hostility	Vigor	Fatigue	Confusion	Total Mood Score
BA	PRE	9.0 ± 6.8	6.1 ± 9.2	6.2 ± 4.1	23.8 ± 5.0	5.4 ± 6.0	7.7 ± 6.1	10.6 ± 27.0
	MID	8.1 ± 5.9	5.2 ± 8.9	5.6 ± 4.0	25.1 ± 5.4	4.0 ± 4.4	6.8 ± 4.3	4.5 ± 27.3
	POST	7.2 ± 4.7	4.7 ± 6.9	4.9 ± 3.7	24.2 ± 6.1	4.7 ± 6.9	6.4 ± 4.1	2.6 ± 23.9
PL	PRE	8.8 ± 5.7	7.0 ± 8.7	6.0 ± 4.9	21.8 ± 5.7	5.4 ± 4.1	8.6 ± 5.4	13.9 ± 25.7
	MID	9.6 ± 6.6	5.8 ± 6.9	7.0 ± 4.6	22.0 ± 6.6	5.0 ± 4.7	9.1 ± 5.7	14.6 ± 28.1
	POST	8.3 ± 5.3	5.7 ± 6.7	5.7 ± 4.4	21.3 ± 7.3	5.7 ± 6.7	8.3 ± 5.3	12.1 ± 25.1

BA = β-alanine; PL = Placebo. All results are presented as mean ± SD.

**Table 2:** Performance Assessments.

Group	Time	Body Mass (kg)	Hand Grip (kg)	Sit-to-Stand (s)	Peak Power (w)	Mean Power (w)	Fatigue Rate (%)
**BA**	PRE	80.5 ± 20.0	32.2 ± 15.6	8.5 ± 2.6	409.7 ± 160.8	368.6 ± 140.3	77.8 ± 13.0
	MID	80.3 ± 19.9	32.6 ± 15.4	7.9 ± 2.3	429.0 ± 157.0	376.8 ± 127.2	75.8 ± 13.7
	POST	80.6 ± 20.3	33.3 ± 15.1	7.6 ± 2.3	409.3 ± 141.4	371.0 ± 130.5	79.7 ± 11.3
**PL**	PRE	69.7 ± 12.6	24.1 ± 9.9	9.4 ± 2.8	332.7 ± 105.1	302.6 ± 95.1	78.4 ± 10.4
	MID	69.8 ± 12.3	24.5 ± 9.2	8.8 ± 2.6	336.2 ± 106.7	311.9 ± 97.0	80.7 ± 9.5
	POST	69.9 ± 12.2	24.8 ± 9.4	8.3 ± 2.7	362.3 ± 117.2	330.6 ± 104.3	79.3 ± 11.4

BA = β-alanine; PL = Placebo. All results are presented as mean ± SD.

## Data Availability

The data presented in this study are available on request from the corresponding author.
